# STRUCTURE PLOT: a program for drawing elegant STRUCTURE bar plots in user friendly interface

**DOI:** 10.1186/2193-1801-3-431

**Published:** 2014-08-13

**Authors:** Ramesh Krishnan Ramasamy, Sumathy Ramasamy, Bharat Bushan Bindroo, V Girish Naik

**Affiliations:** Molecular Biology Laboratory-1, Host Plant Improvement, Central Sericultural Research and Training Institute, Srirampura, Manandavadi Road, Mysore, 570008 India; Bioinformatics Centre, Central Sericultural Research and Training Institute, Srirampura, Manandavadi Road, Mysore, 570008 India

**Keywords:** DISTRUCT, CLUMPP, STRUCTURE HARVESTER, R program, Shiny

## Abstract

**Background:**

Understanding structure of the population is one of the major objective of many genetic studies. The program STRUCTURE is commonly used to infer population structure using multi-locus genotype data. However, a tool with graphical-user interface is currently not available to visualize STRUCTURE bar plots.

**Results:**

We introduce STRUCTURE PLOT, a program for drawing STRUCTURE bar plots. The program generates publication ready, aesthetic STRUCTURE bar plots by using individual Q matrix from STRUCTURE or CLUMPP output. The program is very simple to use and includes variety of options like sorting bar by original order or by K, and selection of colors from R colors or RColorBrewer palette. Individual or population labels can be printed below or above the plot in any angle. Size of the graph and label can be defined, and option is provided to save plot in variety of picture formats in user defined resolution.

**Conclusion:**

The program is implemented as a web application for online users and also as a standalone shiny application. Web application is compatible to majority of leading web browsers and standalone version can be launched using a simple R command. The program can be freely accessed at http://btismysore.in/strplot.

## Introduction

The program STRUCTURE (Pritchard et al. 2000) is one of the widely used genetic analysis software over the last decade to infer population structure and gene flow by using multi-locus genotypic data. STRUCTURE implements model based method to assign each individual to one assumed population (K) or more than one population, if it is an admixture. Estimating suitable assumed population size for the dataset is usually based on maximum likelihood (LnPD) value inferred from STRUCTURE run or delta K (Δ*k*) value (Evanno et al. 2005). STRUCTURE HARVESTER (Earl 2012) program was designed to carry out downstream processing of STRUCTURE results to calculate Evanno’s Δ*k* value and prepares input file for CLUMPP program (Jakobsson and Rosenberg 2007). CLUMPP program permutes replicated runs of STRUCTURE software to find a close match among iterated runs. CLUMPP results are used to generate bar graphs using DISTRUCT (Rosenberg 2004) program. DISTRUCT is a standalone program without Graphical User Interface (GUI), and the drawing parameters needs to be defined in a separate file. DISTRUCT returns the result file in postscript format and users have to install third-party software to convert postscript to the graphical format. To overcome these limitations, we introduce a user-friendly program – ‘STRUCTURE PLOT’ to draw elegant bar plots with graphical user interface.

### Functionality description

**Figure 1:**
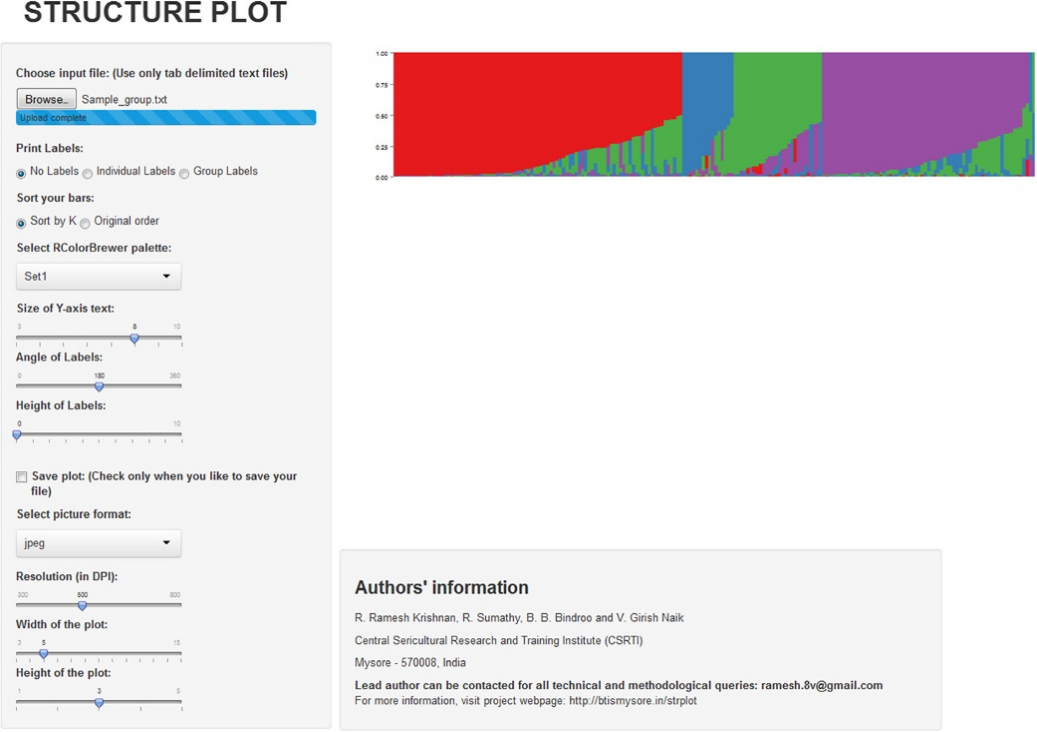
**Screen shot of STRUCTURE PLOT standalone application illustrate the options provided in the program.**

The program is implemented as a web application with user friendly interface also as a shiny (http://www.rstudio.com/shiny/) standalone application. The program accepts individual Q matrix as input file and plot settings can be adjusted using a graphical interface (Figure 1). R is used as scripting language, and ggplot2 package (Wickham 2009) is used for plotting bar graphs. Any suitable colour palette can be selected from RColorBrewer (Neuwirth 2007) or custom colours can be passed to each K. Users can simply copy the individual Q matrix from STRUCTURE or CLUMPP output and used as input file for STRUCTURE PLOT. For a demo, a sample dataset with 202 diverse coconut genotypes sampled by Krishnan et al. (2014) was provided in the webpage. Bars can be drawn in the original order, or it can be sorted by Q. Bar plots are displayed on the same page instead of separate result page. Therefore, the user can play around with settings and visualise the changes in the plot. The program is capable of printing individual or population labels below or above the plot in any angle. Shiny application is built in reactive programming environment, therefore, results can be viewed instantly while adjusting plotting parameters. Options are provided to change the dimensions of the graph and size of the axis labels. Plots can be downloaded in a variety of picture formats in user defined resolution. STRUCTURE PLOT web application can be accessed from any computer with internet connectivity and compatible to majority of the leading web browsers. Shiny application can be launched from R console by using a simple command, and all the parameters can be defined using a graphical interface. We are intended to provide a variety of plotting options in coming days.
